# Comparison of physician- and self-assessed pubertal onset in Japanese children

**DOI:** 10.3389/fped.2023.950541

**Published:** 2023-03-21

**Authors:** Mayako Saito-Abe, Minaho Nishizato, Kiwako Yamamoto-Hanada, Liming Yang, Maki Fukami, Yoshiya Ito, Kenji Ihara, Atsushi Iwabuchi, Shingo Okamoto, Yasuhiro Naiki, Yukihiro Ohya, Reiko Horikawa

**Affiliations:** ^1^ Medical Support Center for the Japan Environment and Children's Study, National Center for Child Health and Development, Tokyo, Japan; ^2^ The Red Cross Hokkaido College of Nursing, Division of Clinical Medicine, Hokkaido, Japan; ^3^ Department of Pediatrics, Faculty of Medicine, Oita University, Oita, Japan; ^4^ Department of Pediatrics, Faculty of Medicine, University of Tsukuba, Ibaraki, Japan; ^5^ Okamoto Internal Medicine and Pediatrics Clinic, Nara, Japan; ^6^ Endocrinology and Metabolism Division, National Center for Child Health and Development, Tokyo, Japan

**Keywords:** epidemiology, self-assessment, tanner stage, pubertal development scale, puberty onset

## Abstract

**Introduction:**

Physical examinations to assess pubertal development are challenging in large epidemiological surveys. This study aimed to assess the reliability of judgment of pubertal onset in Japanese children by the original pubertal self-assessment sheet.

**Methods:**

A total of 144 children aged 10 or 12 years were recruited between March 2019 and September 2020 from the pediatric endocrine outpatient clinics of participating institutions. Agreement between the physician- and participantassessed pubertal onsets was determined using unweighted kappa (UK) and Gwet's agreement coefficient (AC1).

**Results:**

The physician's assessment of pubertal onset was in slight agreement with that of the self-assessment sheet in 10-year-old boys (UK: 0.23 and AC1: 0.14), whereas the agreement between the physician's assessment and self-assessment sheet results was good and the physician's assessment was fair (UK: 0.64 and AC1: 0.94) in 12-year-old boys. The physician's assessment of pubertal onset were in good and moderate agreement with the self-assessment sheet in 10-year-old girls (UK/AC1: 0.74/0.78, respectively). In 12-year-old girls, although it showed poor agreement with UK (0.46), there was a very good agreement with AC1 (0.88).

**Conclusions:**

Although self-assessment of breast development was in good agreement with that of the physician's assessment for determining pubertal onset in girls, large-scale epidemiological studies are difficult to conduct for adolescent boys, especially for those in the early pubertal stage.

## 1. Introduction

Puberty—a transition period from childhood to adolescence—involves significant physiological changes and sexual maturation, and pubertal assessment is an essential component of epidemiological studies. For example, we conducted the Japan Environment and Children's Study (JECS) (a nationwide, multicenter, prospective, birth cohort study) that enrolled approximately 100,000 mothers and their children since 2011 (1, 2), and environmental substances that impact pubertal development are a crucial issue examined in our cohort study. In addition to the questionnaire survey for all participants, JECS conducts a medical survey of some randomly selected participants. We considered assessing the pubertal onset in the medical survey conducted for the participants aged 10 and 12, in order to examine our endocrinological research hypotheses.

The Tanner sexual maturity scale (3, 4) is the gold standard for assessing sexual maturation and the onset of puberty in children (5). This scale requires that the child is undressed for a physical examination, which is conducted by healthcare professionals. The JECS study had considered conducting a physical examination to determine the onset of puberty using the Tanner scale and concluded that it would be ethically challenging and not feasible. Therefore, alternatives to physical examination were needed to assess the onset of puberty. According to the previous review study, young people thought that self-assessment was more acceptable than the assessment by physicians (6). Other epidemiological studies employed self-assessment using pictures from the Tanner sexual maturity scale as a method to determine the pubertal stage without the need for examination by a physician (7–9). However, the use of these vivid pictures or drawings for children may not be welcomed by caregivers. Previous studies using the Tanner pubertal questionnaire including pictures reported a total refusal rate of 20%, and the authors speculated that the direct approach used in the assessment may be objectionable to participants (10, 11). We should also consider that the validity of self-assessment of puberty stage have also been controversial, with one literature reporting high rates of agreement with physician's assessment in adolescents of both sexes (12), but some reports of lower rates of agreement in boys (13, 14). In addition, to the best of our knowledge, there are no reports of similar studies in the Japanese adolescent population. Therefore, we developed a pubertal self-assessment sheet for Japanese adolescents focusing focusing on a testicular volume of 4 ml in boys and Tanner stage 2 for breast development in girls, both of which are widely accepted as the gold standard of pubertal onset in the clinical setting. Importantly, we created original pictures tailored to the Japanese population in this sheet.

In this study, we aimed to assess the reliability of judgment of pubertal onset by children aged 10 and 12 years using the pubertal self-assessment sheet, and we compared the findings with those of a physician's assessment as the gold standard.

## 2. Material and methods

### 2.1. Study design, setting, and participants

This was a cross-sectional study that included children who were recruited between March 2019 and September 2020 at the pediatric endocrine outpatient clinics of the National Center for Child Health and Development (Tokyo), Kitami Red Cross Hospital (Hokkaido), Oita University Hospital (Oita), and Tsukuba University Hospital (Ibaraki). The eligibility criteria for the participants were as follows: 10- and 12-year-old boys and girls who were Japanese native speakers, had no difficulty in answering the questionnaire or self-assessment sheet by themselves, and were not visiting the clinic for the first time. Participants with underlying endocrine diseases were not excluded. The institutional review board of each institution approved the study protocol, and informed assent and consent were obtained from the children (approval numbers: 2,986 for National Center for Child Health and Development, 30–318 for Kitami Red Cross Hospital, 1,594 for Oita University Hospital, and H30–345 for Tsukuba University Hospital).

### 2.2. Study procedures and clinical assessments

The onset of puberty was defined as testicular enlargement of 4 ml in boys and Tanner stage 2 breast development in girls.

The participants were recruited while they were waiting in the outpatient clinic, and those who agreed to participate in the study completed the questionnaires before or after the regular consultations. Subsequently, the pediatric endocrinologist who examined the participants described the participants' pubertal development (Tanner stage for boys and girls and testicular volume for boys) on a physician check sheet. The pubertal assessments were conducted as part of the routine clinical examination during the patient's visit to the clinic. Simultaneously, the participants were instructed to complete the pubertal self-assessment sheet in a space separated by a curtain in the medical examination room.

We did not collect any personal information, including underlying medical conditions, because it was not essential to achieve the purpose of this study.

### 2.3. Pubertal self-assessment sheet

Separate pubertal self-assessment sheets were developed for boys and girls. For boys, left and right testicular volumes were described in a range of 25 levels (Supplementary Appendix S1) based on the comparison with the Okamoto testicular volume self-assessment sheet, which was developed by Shingo Okamoto for the screening of hypogonadism in 15-year-old boys. The pubertal self-assessment sheet for girls included an illustration of Tanner's stage 2 breast development and an explanation that puberty has begun if that stage is reached (Supplementary Appendix S2). The illustration of Tanner's stages was originally developed in full color by a certified medical illustrator to facilitate easier comprehension by children. For both boys and girls, the answer “I don’t know/I don’t want to answer” was considered as missing data.

### 2.4. Statistical analysis and sample size calculation

Cohen's kappa and Gwet's AC1 statistics with 95% confidence intervals (CIs) were calculated to evaluate the inter-rater agreement. Participants with results without missing values and those who answered “I don’t know/I don’t want to answer” to the applicable items were included in the analysis.

Cohen's kappa and Gwet's AC1 values were interpreted as follows: <0.20, poor; 0.21–0.40, fair; 0.41–0.60, moderate; 0.61–0.80, good; and 0.81–1.00, very good agreement (15, 16). All statistical analyses were performed using R software version 4.0.3 (Institute for Statistics and Mathematics, Vienna, Austria; www.r-project.org).

To calculate the sample size, a clinically acceptable target value (expected value) of 0.8 and a threshold value (worst value to be rejected by the null hypothesis) of 0.3 were set as acceptable kappa coefficients based on previous reports and consultation with biostatisticians. The assumed rates of onset of puberty at ages 10 and 12 years for Japanese boys and girls were based on the data of previous studies. Matsuo et al. reported that 25% and 90% of boys presented with testicular enlargement of ≥4 ml at the ages of 10 and 12 years, respectively (17). Tanaka et al. reported that 50% and 90% of girls presented with Tanner stage ≥ 2 for breast development at the ages of 9 years and 11 years and 9 months, respectively (18). A total of 120 participants, including 17 girls aged 10 years, 41 girls aged 12 years, 21 boys aged 10 years, and 41 boys aged 12 years, were deemed necessary for the final analyses. To allow for the possibility of missing data, the recruitment target was set at 130 participants.

## 3. Results

A total of 168 participants were enrolled, and 144 were included in the study. The consent acquisition rates were 95.8% and 88.9% for 10-year-old boys and girls, respectively, and 77.6% and 88.1% for 12-year-old boys and girls, respectively (data not shown). We used the data of 122 participants for the final calculation of Cohen's kappa and Gwet's AC1, excluding the missing values, as shown in Table 1. The rate of missing values, including those for “I don’t know/I don’t want to answer,” for each question were dependent on the age and sex. In the self-assessment sheet, girls demonstrated a higher rate of missing data than boys at both ages (29.2% vs. 8.7% at 10 years; 17.3% vs. 8.9% at 12 years).

**Table 1:** Participants’ response status to the self-assessment sheet and the number of participants included in the kappa and Gwas's AC1 calculation.

	10-year-old participants		12-year-old participants		All
	Boys	Girls	Boys	Girls	
**Calculated sample size, *n***	**21**	**17**	**41**	**41**	**120**
Recruited, *n*	23	24	45	52	144
I don’t know/I don’t want to answer, *n* (%)	2 (8.7%)	7 (29.2%)	4 (8.9%)	9 (17.3%)	25 (17.4%)
Missing of physician assessment, *n* (%)	0 (0.0%)	0 (0.0%)	1 (2.2%)	2 (3.8%)	3 (2.1%)
**Included in the kappa and Gwas's AC1 calculation, *n***	**21**	**17**	**41**	**42**	**122**

Table 2 shows the comparison of pubertal onset in boys and girls based on the physician's assessment and self-assessment sheet. The testicular volumes of ≥4 ml were 34.8% and 91.1% by physician's assessment and 60.9% and 84.4% by self-assessment for boys aged 10 and 12 years, respectively. The rates of agreement among those ways of assessment were 52.2% and 86.6%, respectively. Tanner stage of ≥2 for breast development was 58.3% and 92.3% by physician's assessment and 45.8% and 71.2% by self-assessment for girls aged 10 and 12 years, respectively. The rates of agreement among those ways of assessmentwere 62.5% and 73.0%, respectively.

**Table 2:** ** ** Comparison of pubertal onset in boys and girls based on the physician's assessment and the pubertal self-assessment sheet.

		Physician's assessment					
		Missing^a^	No	Yes	Missing^a^	No	Yes
Boys		10-year-old participants (*n* = 23)			12-year-old participants (*n* = 45)		
Self-assessment sheet	Missing^a^	0	1	1	1	0	3
	No	0	**6 (26.1%)**	1	0	** 2 (4.4%) **	1
	Yes	0	8	**6 (26.1%) **	0	1	**37 (82.2%)**
Girls		10-year-old participants (*n* = 24)			12-year-old participants (*n* = 52)		
Self-assessment sheet	Missing^a^	0	4	3	1	0	8
	No	0	**5 (20.8%) **	1	0	**2 (3.9%) **	4
	Yes	0	1	**10 (41.7%) **	1	0	**36 (69.2%)**

^a^ Includes the response “I don’t know/I don’t want to answer”.

The details of Tanner's pubertal stages in children and the distribution of testicular volume assessed by the physicians are shown in Supplementary Table S1 and Supplementary Figure S3.

Table 3 shows the agreement between physician-assessed and self-reported pubertal onset, which was calculated byunweighted kappa (UK) and AC1. Boys aged 10 years did not reach the clinical acceptance threshold-fair agreement based on UK and slight agreement based on AC1. Conversely, in 12-year-old boys, the agreement was good based on UK and very good based on AC1. For girls, although 10-year-old participants demonstrated good agreement in terms of both UK and AC1, there was a difference among 12-year-old participants. UK revealed a moderate agreement and a very good agreement with AC1.

**Table 3:** Agreement of the determination of pubertal onset based on the assessment by the physician, self-assessment sheet.

	10-year-old participants		12-year-old participants	
	Value (95% CI)	Interpretation	Value (95% CI)	Interpretation
Boys	(*n* = 21)		(*n* = 41)	
Kappa	0.23 (−0.09, 0.54)	Fair	0.64 (0.18, 1.00)	Good
Gwet's AC1	0.14 (−0.32, 0.61)	Poor	0.94 (0.86, 1.00)	Very Good
Girls	(*n* = 17)		(*n* = 42)	
Kappa	0.74 (0.41, 1.00)	Good	0.46 (0.04, 0.88)	Moderate
Gwet's AC1	0.78 (0.46, 1.00)	Good	0.88 (0.76, 1.00)	Very Good

CI, confidence interval.

Cohen's kappa and Gwet's AC1 values were interpreted as follows: <0.20, poor; 0.21–0.40, fair; 0.41–0.60, moderate agreement; 0.61–0.80,good; and 0.81–1.00, very good.

## 4. Discussion

In this study, we compared the agreement of pubertal onset in 10- and 12-year-old Japanese children based on the physician's assessment with that based on the newly developed pubertal self-assessment sheet.

The physician's assessment exhibited fair/poor agreement with the self-assessment sheet in 10-year-old boys and good/very good agreement in 12-year-old boys. The results suggested that the determination of pubertal onset in 12-year-old boys was easy because of the significant increase in testicular volume beyond the cutoff value of 4 ml and given that accurate assessment of the testicular volume in 10-year-old boys was challenging. The Okamoto testicular volume self-assessment sheet used for the self-assessment method in the present study was originally validated for 15-year-old children with gonadal dysgenesis; the present study results indicate that this method might not be suitable for self-assessment of pubertal onset. In girls, the agreement between the physician's assessment and the pubertal self-assessment sheet was good in the 10-year-old group. However, the agreement was moderate between these assessments with the kappa statistic for the 12-year-old group. The kappa is sensitive to the rater's classification probability (19, 20). The extremely low prevalence of prepuberty (7.7%) in the 12-year-old group resulted in a biased kappa statistic. AC1 can overcome this shortcoming and provide a more robust estimation (20). In fact, the moderate result of the kappa statistic was not in accordance with the finding that 69.2% of the girls correctly assessed their puberty using the self-assessment sheet. Conversely, the result of AC1 for the agreement was good. Therefore, the determination of pubertal onset was acceptable using self-assessment sheet methods in girls, especially among those aged 10 years. In general, the girls were more likely to agree with the physician's diagnosis compared with the boys in the present study, a finding that is consistent with previous reports validating adolescent self-diagnosis (12, 14). The major reason ofthis sexual difference was that breast growth, which was the subject of puberty assessment in girls in the present study, was easier to assess objectively than testicular volume in boys. Regarding the Okamoto testicular volume self-assessment sheet, “knowing what the testes are” is necessary to capture the testes under the epidermis adequately, and the thickness of the skin of the scrotum should be considered. However, children in Japan do not have adequate opportunities to learn about the testes, especially elementary school-aged children.

Overall, our findings suggest that the reliability of self-assessment varies with age, especially in boys. Previous studies, which did not focus on specific ages, suggested that the reliability of self-assessment depends on endpoints and goals (14, 21). Morris et al. examined the correlation between the physician's diagnosis of Tanner stage and testicular volume using a questionnaire in 12–16-year-old boys (22) and found that the Pearson's correlation coefficients were 0.59 for genital development, 0.63 for genital hair distribution, and 0.18 for testicular volume; the authors considered that reaching an agreement between self-assessment and physician's assessment would be difficult even in participants aged >12 years. Rollof et al. examined the correlation between physician- and self-assessed staging of testicular volume using an orchidometer in 10–16-year-old children (23); they found that the rate of agreement was 36% and that the difference was only by one degree in 95% of the assessments. They concluded that pubertal self-assessment including the use of an orchidometer for boys, albeit a useful method to determine the exact pubertal onset, should be performed by a trained professional. Rasmussen et al. concluded that pubertal assessment performed by the child and the parent among 7–14-year-old children were not reliable measures of exact pubertal staging and should be corroborated by physical examination (21). However, the authors also stated that self-assessment could be sufficiently accurate for a simple distinction between prepuberty and puberty in large epidemiologic studies.

The strength and significance of this study were that this was the first study to assess the reliability of self-assessment of pubertal onset using the original puberty evaluation sheet among Japanese children. Although pubertal assessment is an essential component of epidemiological studies to assess the health of children, performing physical examinations of the participants was not usually feasible. This issue was our primary focus, and this cross-sectional study could yield important insights.

## 5. Limitations of the studies

Several limitations should be considered in the interpretation of the study findings. First, the participants were patients with underlying endocrine diseases, including those with early- and late-onset puberty and data on the underlying diseases were not collected. The impact of this limitation on the results was considered low because the only study endpoint was the agreement between self-assessment and the physician's assessment for the onset of puberty. Additionally, there was no significant difference in pubertal onset between the present study cohort and previously reported cohorts of the same age groups. Second, we recruited patients during regular outpatient visits and were unable to examine test–retest reliability because of the infrequency of each patient's visits and the possibility that the endpoints may change over time. Third, we only assessed the children who were aged 10 and 12 years and thus did not collect the data on other age groups. Future studies should include a study population with a wider age range.

## 6. Conclusions

In conclusion, this study indicated that the assessment of pubertal onset by self-assessment of testicular volume in boys immediately after the start of puberty was difficult. In contrast, self-assessment of breast development in girls was in good agreement with the physician's assessment. Adolescent studies in large-scale epidemiological studies remain challenging, especially for boys. Acceptable and valid assessment methods for puberty in both sexes would make epidemiological studies more feasible at adolescent age. Further developmental study of self-pubertal assessment methods is needed.

## Data Availability

The datasets presented in this article are not readily available due to IRB restrictions. Requests to access the datasets should be directed to MN, nishizato-m@ncchd.go.jp.
