# Supplementary material for: Comparison of physician- and self-assessed pubertal onset in Japanese children
Source: Front Pediatr. 2023 Mar 21;11:950541. doi: 10.3389/fped.2023.950541 (PMC10070871; doi:10.3389/fped.2023.950541)
Supplement: Supplementary file 1 [file Table1.docx]

Table S1. Tanner’s pubertal stages of children as assessed by the physicians.

| Tanner stage (physician’s assessment), n (%) | | | | | |
| --- | --- | --- | --- | --- | --- |
|  | | 10-year-old participants | | 12-year-old participants | |
|  | Boys | Girls | Boys | Girls |  |
| Breast | Stage 1 | - | 10 (41.7%) | - | 2 (3.8%) |
|  | Stage 2 | - | 8 (33.3%) | - | 8 (15.4%) |
|  | Stage 3 | - | 5 (20.8%) | - | 22 (42.3%) |
|  | Stage 4 | - | 1 (4.å2%) | - | 17 (32.7%) |
|  | Stage 5 | - | 0 (0.0%) | - | 1 (1.9%) |
|  | Missing | - | 0 (0.0%) | - | 2 (3.8%) |
|  | ≥Stage 2 | - | 14 (58.3) | - | 48 (92.3%) |
| Pubic Hair | 1 | 19 (82.6%) | 19 (79.2) | 25 (55.6%) | 20 (38.5%) |
|  | 2 | 3 (13.0%) | 4 (16.7%) | 9 (20.0%) | 8 (15.4%) |
|  | 3 | 1 (4.3%) | 1 (4.2%) | 7 (15.6%) | 10 (19.2%) |
|  | 4 | 0 (0.0%) | 0 (0.0%) | 2 (4.4%) | 9 (17.3%) |
|  | 5 | 0 (0.0%) | 0 (0.0%) | 0 (0.0%) | 0 (0.0%) |
|  | Missing | 0 (0.0%) | 0 (0.0%) | 2 (4.4%) | 5 (9.6%) |
| Genitals | 1 | 16 (69.6%) | - | 5 (11.1%) | - |
|  | 2 | 6 (26.1%) | - | 16 (35.6%) | - |
|  | 3 | 1 (4.3%) | - | 19 (42.2%) | - |
|  | 4 | 0 (0.0%) | - | 3 (6.7%) | - |
|  | 5 | 0 (0.0%) | - | 0 (0.0%) | - |
|  | Missing | 0 (0.0%) | - | 2 (4.4%) | - |
| Testicular volume | <4 mL | 15 (65.2%) | - | 3 (6.7%) | - |
|  | ≥4 mL | 8 (34.8%) | - | 41 (91.1%) | - |
|  | Median (IQR), mL | 3 (2) | - | 8 (5.3–11.5) | - |
|  | Missing | 0(0.0%) | - | 1(2.2%) | - |

 IQR; interquartile range

Tanner stage 2 or higher breast development was reported in 58.3% and 92.3% of the 10- and 12-year-old girls, respectively. The median testicular volumes as assessed by the physicians were 3 and 8 mL in 10- and 12-year-old boys, respectively. We found that 34.8% and 91.1% of the 10- and 12-year-old boys had testicular volumes of ≥4 mL. The Doctor-assessment testis volume distribution is shown in Figure S3.
