# Supplementary material for: Comparison of physician- and self-assessed pubertal onset in Japanese children
Source: Front Pediatr. 2023 Mar 21;11:950541. doi: 10.3389/fped.2023.950541 (PMC10070871; doi:10.3389/fped.2023.950541)
Supplement: Supplementary file 2 [file Presentation1.pptx]

## Slide 1
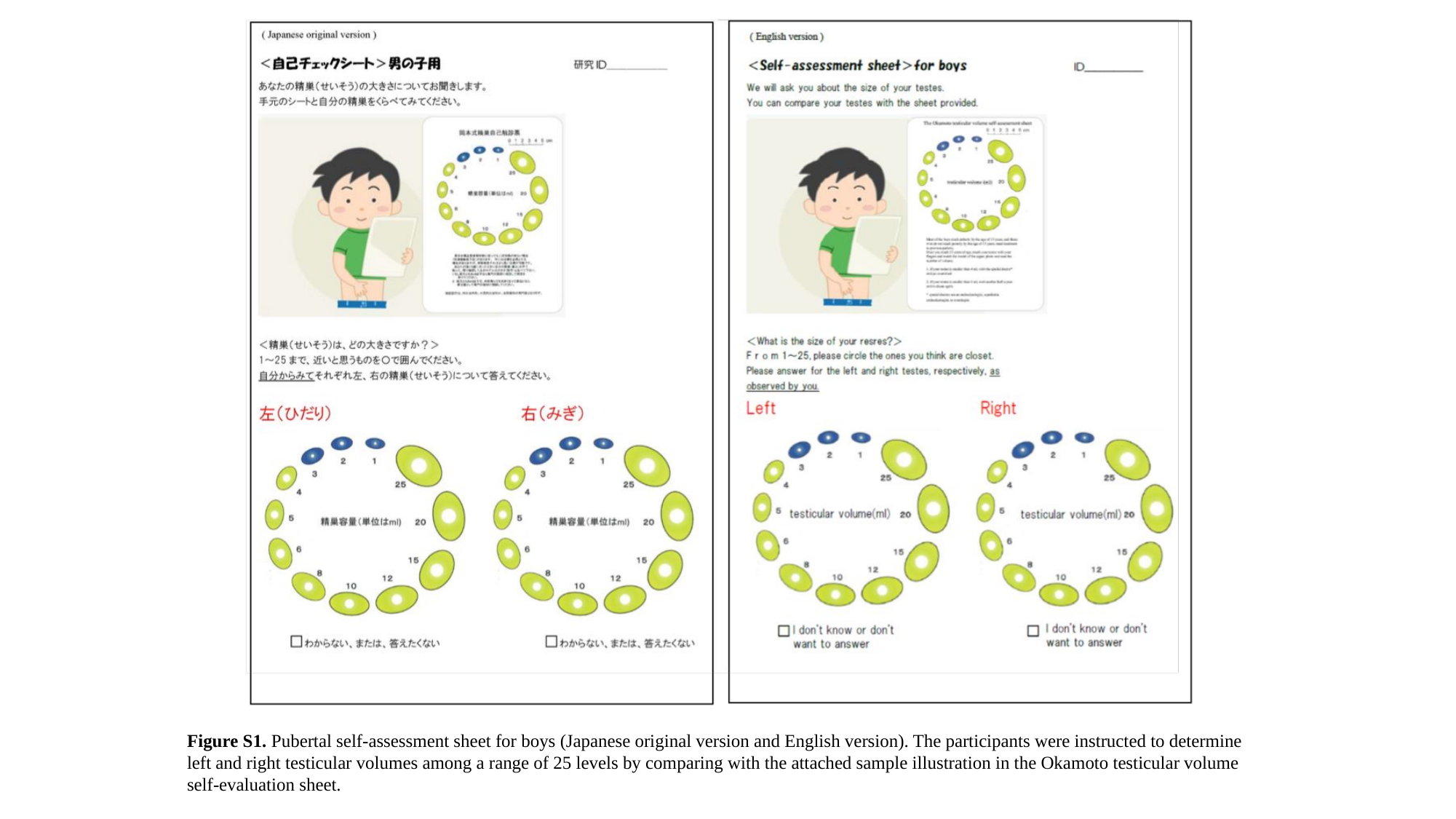

Figure S1. Pubertal self-assessment sheet for boys (Japanese original version and English version). The participants were instructed to determine left and right testicular volumes among a range of 25 levels by comparing with the attached sample illustration in the Okamoto testicular volume self-evaluation sheet.
