# Supplementary material for: Comparison of physician- and self-assessed pubertal onset in Japanese children
Source: Front Pediatr. 2023 Mar 21;11:950541. doi: 10.3389/fped.2023.950541 (PMC10070871; doi:10.3389/fped.2023.950541)
Supplement: Supplementary file 3 [file Presentation2.pptx]

## Slide 1
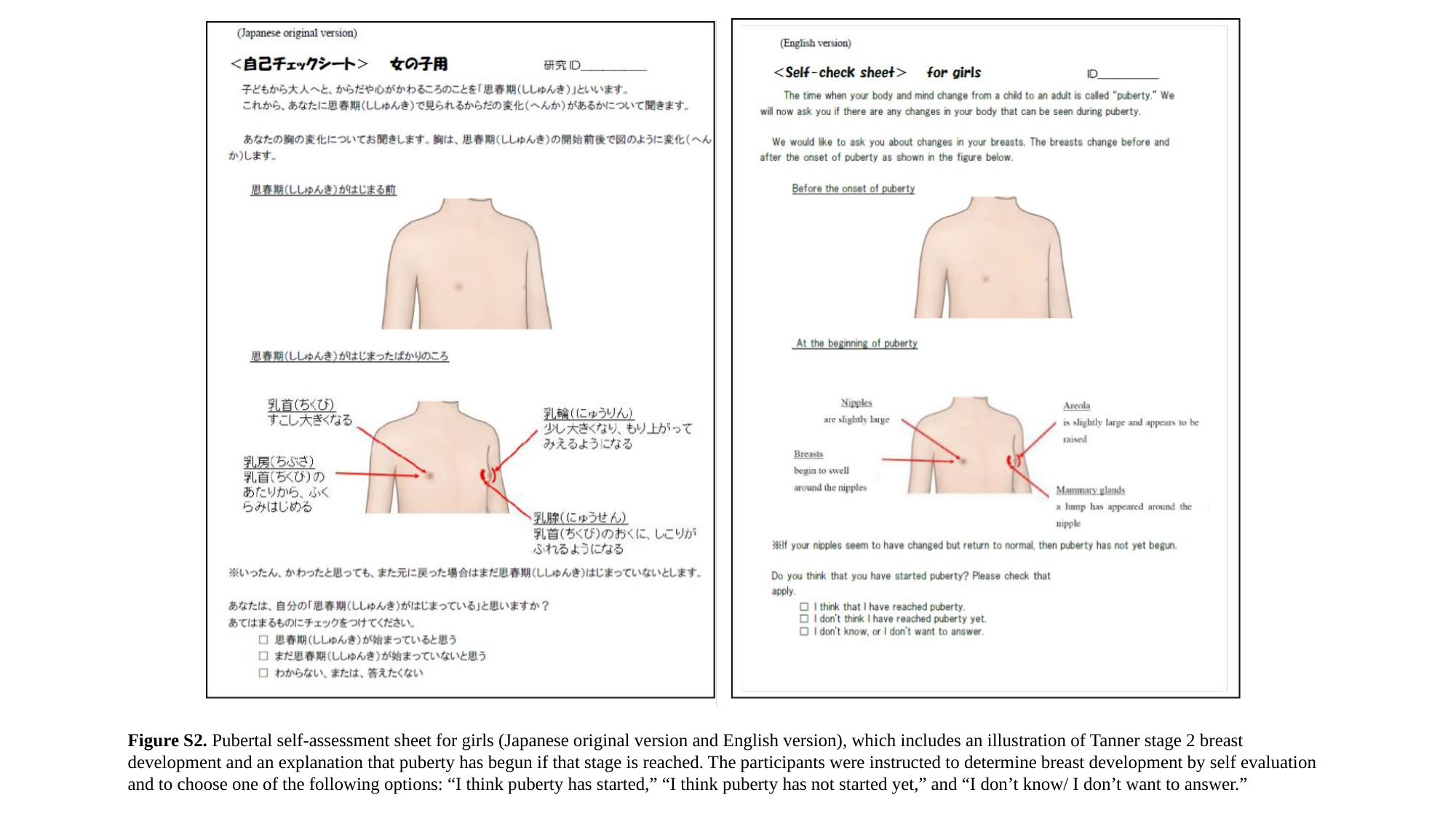

Figure S2. Pubertal self-assessment sheet for girls (Japanese original version and English version), which includes an illustration of Tanner stage 2 breast development and an explanation that puberty has begun if that stage is reached. The participants were instructed to determine breast development by self evaluation and to choose one of the following options: “I think puberty has started,” “I think puberty has not started yet,” and “I don’t know/ I don’t want to answer.”
