# Supplementary material for: Comparison of physician- and self-assessed pubertal onset in Japanese children
Source: Front Pediatr. 2023 Mar 21;11:950541. doi: 10.3389/fped.2023.950541 (PMC10070871; doi:10.3389/fped.2023.950541)
Supplement: Supplementary file 4 [file Presentation3.pptx]

## Slide 1
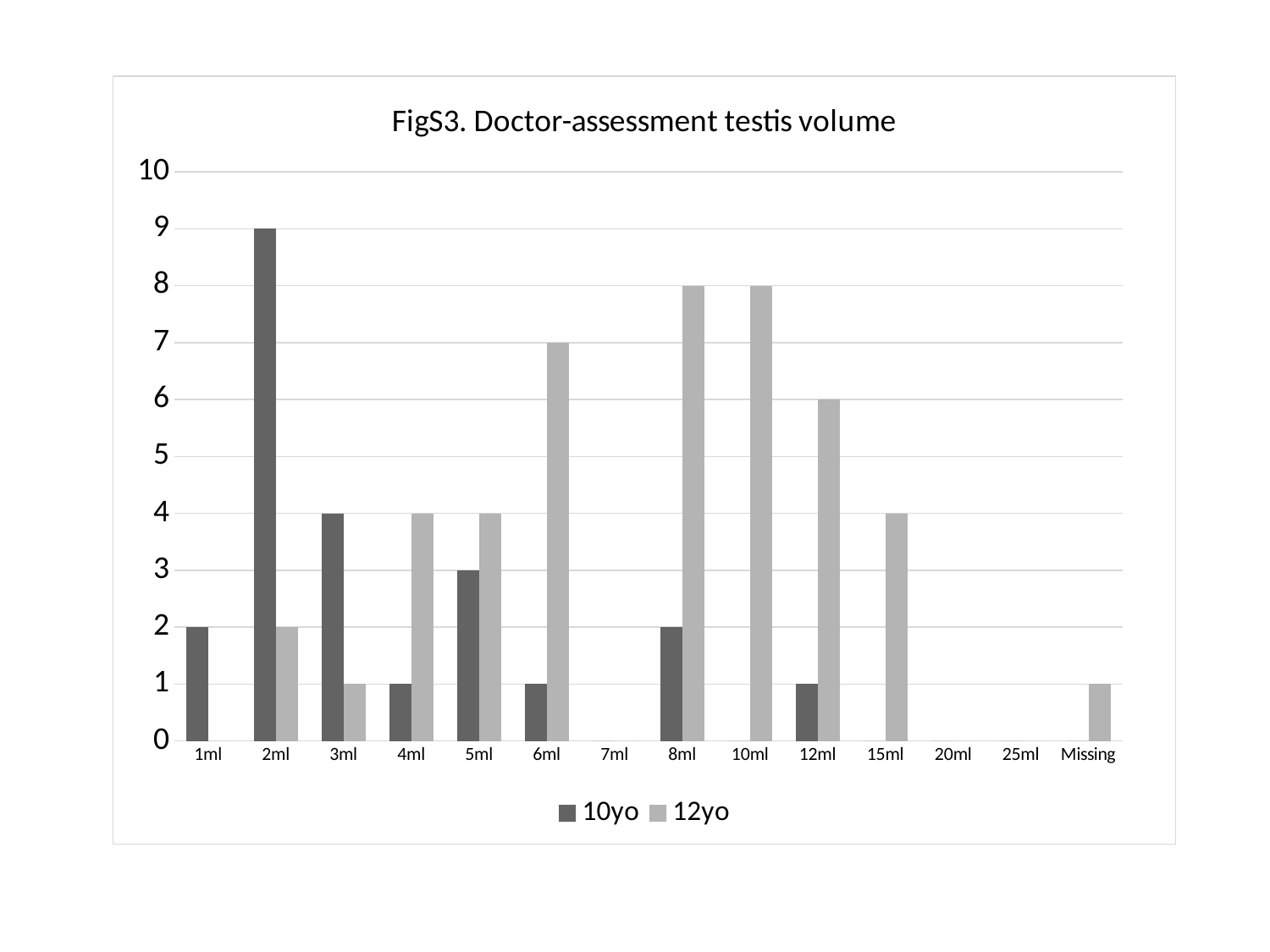

### Chart: FigS3. Doctor-assessment testis volume
| Category | 10yo | 12yo |
|---|---|---|
| 1ml | 2.0 | 0.0 |
| 2ml | 9.0 | 2.0 |
| 3ml | 4.0 | 1.0 |
| 4ml | 1.0 | 4.0 |
| 5ml | 3.0 | 4.0 |
| 6ml | 1.0 | 7.0 |
| 7ml | 0.0 | 0.0 |
| 8ml | 2.0 | 8.0 |
| 10ml | 0.0 | 8.0 |
| 12ml | 1.0 | 6.0 |
| 15ml | 0.0 | 4.0 |
| 20ml | 0.0 | 0.0 |
| 25ml | 0.0 | 0.0 |
| Missing | 0.0 | 1.0 |
